# Non-Canonical Compartmentalization of DROSHA Protein at the Golgi Apparatus: miRNA Biogenesis-Independent Functionality in Human Cancer Cells of Diverse Tissue Origin

**DOI:** 10.3390/ijms26199319

**Published:** 2025-09-24

**Authors:** Eleni I. Theotoki, Panos Kakoulidis, Kostas A. Papavassiliou, Konstantinos-Stylianos Nikolakopoulos, Eleni N. Vlachou, Efthimia K. Basdra, Athanasios G. Papavassiliou, Ourania E. Tsitsilonis, Gerassimos E. Voutsinas, Athanassios D. Velentzas, Ema Anastasiadou, Dimitrios J. Stravopodis

**Affiliations:** 1 Section of Cell Biology and Biophysics, Department of Biology, School of Science, National and Kapodistrian University of Athens (NKUA), 157 01 Athens, Greece; elthk@biol.uoa.gr (E.I.T.); ksnikolakop@biol.uoa.gr (K.-S.N.); eleni.vlachou.2002@gmail.com (E.N.V.); tveletz@biol.uoa.gr (A.D.V.); 2 Center of Basic Research, Biomedical Research Foundation of the Academy of Athens (BRFAA), 115 27 Athens, Greece; anastasiadou@bioacademy.gr; 3 Department of Informatics and Telecommunications, School of Science, National and Kapodistrian University of Athens (NKUA), 157 01 Athens, Greece; pkakoulidis@di.uoa.gr; 4 First University Department of Respiratory Medicine, “Sotiria” Chest Hospital, Medical School, National and Kapodistrian University of Athens (NKUA), 115 27 Athens, Greece; konpapav@med.uoa.gr; 5 Department of Biological Chemistry, Medical School, National and Kapodistrian University of Athens (NKUA), 115 27 Athens, Greece; ebasdra@med.uoa.gr (E.K.B.); papavas@med.uoa.gr (A.G.P.); 6 Section of Animal and Human Physiology, Department of Biology, School of Science, National and Kapodistrian University of Athens (NKUA), 157 01 Athens, Greece; rtsitsil@biol.uoa.gr; 7 Laboratory of Molecular Carcinogenesis and Rare Disease Genetics, Institute of Biosciences and Applications, National Center for Scientific Research (NCSR) “Demokritos”, 153 10 Athens, Greece; mvoutsin@bio.demokritos.gr

**Keywords:** cancer, DROSHA, Golgi apparatus, malignancy grade, metastasis, miRNA, p53, RNAi

## Abstract

DROSHA protein is widely known for its essential role in the microRNA (miRNA/miR) biogenesis pathway where, together with its co-factor DGCR8, it forms the “Microprocessor” complex and catalyzes the primary miRNA (pri-miRNA) processing in the nucleus. Nevertheless, DROSHA also seems to participate in several miRNA-independent cellular mechanisms, such as transcriptional regulation, RNA processing and genome integrity maintenance. Hence, the present study aims to further investigate novel miRNA-independent activities of DROSHA protein, with potentially regulatory roles in the oncogenesis of human cancer cells. Our results reveal a new, strong profile of microprocessor-independent DROSHA localization at the Golgi apparatus in several human cancer cell lines of different tissue origin, with hepatic carcinoma, thyroid cancer, urothelial bladder cancer, colon carcinoma and melanoma being the cellular model systems herein examined. Notably, oncogenic activity, malignancy grade and metastatic capacity are shown to be strongly associated with DROSHA’s compartmentalization at Golgi, a phenotype that does not seem to rely on p53 protein’s functionality. Taken together, through employment of advanced confocal laser scanning microscopy (CLSM) and molecular modeling, we herein unveil the ability of DROSHA, but not AGO2 and DICER, to reside at Golgi, where DROSHA can physically interact with the GM130 Golgi-specific component, thus indicating DROSHA’s engagement in non-canonical and miRNA-independent—but also Golgi apparatus-dependent—novel mechanisms that can be tightly coupled with malignancy dynamics and beneficially utilized as potential biomarkers and therapeutic targets for human cancer.

## 1. Introduction

DROSHA protein is best known for its fundamental role in the biogenesis of microRNA species (miRNAs/miRs), a group of endogenous, non-coding, single-stranded RNAs of approximately 22 nucleotides in length that are critically involved in gene expression regulation, directing post-transcriptional repression via complementarity to target transcripts [1,2,3,4,5]. Specifically, DROSHA mediates the nuclear processing of primary miRNA (pri-miRNA) generated by RNA polymerase II (RNAPII), yielding the precursor miRNA (pre-miRNA) form of approximately 70 nucleotides in length, which is then exported into the cytoplasm by the Ran-GTP-dependent transporter Exportin-5 (Supplementary Figure S1). In the cytoplasm, pre-miRNA is further processed by the RNase III enzyme DICER to generate mature miRNA, which is then loaded onto RISC (RNA-induced Silencing Complex) to direct post-transcriptional silencing through either mRNA degradation or mRNA translational repression [6,7,8,9].

DROSHA is a large nuclear protein (~160 kDa in humans) that is conserved exclusively and only in the animal kingdom [10,11,12]. Human DROSHA protein contains two RNase III domains (RIIIDa and RIIIDb) and one double-stranded RNA binding domain (dsRBD). The two RIIIDs form an intra-molecular dimer, with the C-terminal RIIIDb cleaving the 5′-strand and the RIIIDa cleaving the 3′-strand of the substrate’s hairpin [2,13,14,15]. In addition, DROSHA carries a Proline (P)-rich region, as well as a Serine/Arginine (S/A) region at the N-terminal area, which seems to be important for nuclear localization of the protein, while the middle region, together with the RIIIDs and dsRBD, is required for the pri-miRNA processing [16].

Because of an inherent insufficiency to bind target substrates on its own, DROSHA needs a co-factor that can recognize pri-miRNA structures [2,17,18,19]. Thereby, another RNA-binding protein (RBP), DGCR8 (DiGeorge Syndrome Critical Region Gene 8), a ubiquitously expressed protein of ~120 kDa containing two dsRBDs and a putative WW (also termed Rsp5/wwp) domain (which typically comprises two signature Tryptophan (W) residues that are spaced 20–23 amino acids apart) [20,21], interacts with DROSHA and forms a functional complex called a “Microprocessor” [17,18]. A microprocessor is a large complex weighing ~650 kDa (in humans) and containing one DROSHA and two DGCR8 protein molecules [16,18]. DROSHA and DGCR8 are both required for miRNA biogenesis, as only their combination and assembly can lead to pri-miRNA processing activity, with DGCR8 offering substrate recognition and DROSHA providing catalytic reaction [2,14,18,22]. Interestingly, there is a cross-regulation between DROSHA and DGCR8; an active microprocessor cleaves *DGCR8* mRNA hairpins produced at the 5′-end, reducing protein levels, while DROSHA is stabilized by DGCR8 via a protein/protein interaction (PPI) mode [1,13,23,24].

However, besides its essential role(s) in the miRNA/RNA interference (RNAi) pathway, DROSHA has been demonstrated to also carry other miRNA-independent functions, being critically involved in the control of transcriptional regulation, RNA processing and genome integrity maintenance [4]. Importantly, DROSHA can destabilize non-canonical, hairpin-containing transcripts, strongly regulating neurogenesis and myelopoiesis, while the DROSHA-mediated control of retrotransposons in human cells has also been reported [25,26,27,28]. Moreover, DROSHA can positively regulate gene expression via association with (proximal) promoter regions, and it is involved in alternative splicing regulation and DNA damage response (DDR)-pathway activation as well [29,30,31,32]. The anti-viral functions and anti-oncogenic properties of DROSHA protein in human cells have also been previously described [13,33,34,35].

The present study aims to further investigate the novel miRNA-independent activities of DROSHA protein, with a potentially regulatory role(s) in the oncogenesis of human cancer cells of diverse tissue origin. The strong localization of DROSHA in unexpected non-canonical, sub-cellular areas, and especially at the Golgi apparatus, in cancer cells likely renders “DROSHA-at-Golgi” a novel, powerful biomarker and a promising drug target for human cancer chemotherapy in clinical practice.

## 2. Results

### 2.1. Non-Canonical DROSHA Distribution in the Cytoplasm of Human Immortalized Cells During Interphase

**Figure 1:**
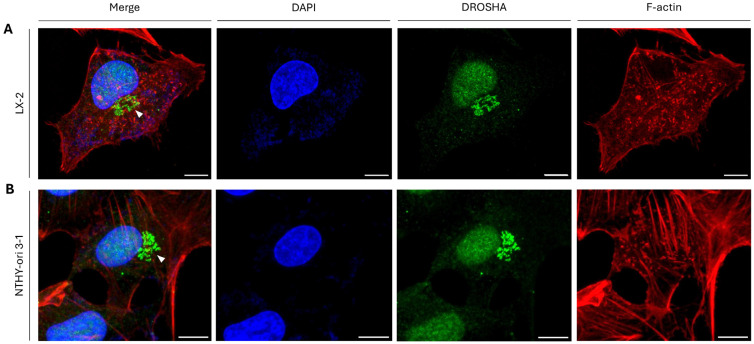
**Sub-cellular distribution of DROSHA protein in human hepatic/liver and thyroid immortalized non-tumorigenic cells.** CLSM immunofluorescence images of (**A**) LX-2 (human stellate hepatic/liver) and (**B**) NTHY-ori 3-1 (human thyroid follicular/epithelial) cells, presenting DROSHA’s sub-cellular distribution (white arrowheads: “DROSHA bodies”). DROSHA protein is visualized in green, F-actin (F: Filamentous) is shown in red and the cell nucleus is depicted in blue (DAPI). Scale bar: 10 μm.

DROSHA protein displays a predominant nuclear distribution mechanistically related to its essential role(s) in miRNA biogenesis pathway. However, our immunofluorescence results derived from LX-2 (human stellate hepatic/liver) cells demonstrated, besides its typical nuclear accumulation, DROSHA’s topology in an unexpected sub-cellular location of the LX-2 cytoplasm during the interphase stage with DROSHA residing in a distinct structure(s) close to the nucleus (Figure 1A).

To investigate whether the obtained phenotype is specific for hepatic/liver cells, we next examined the protein’s distribution pattern in NTHY-ori 3-1 (human thyroid follicular/epithelial) cells. Interestingly, the compartmentalization of DROSHA was not limited to the nucleus, and a cytoplasmic DROSHA immunofluorescence profile could also be asymmetrically detected proximally to the nucleus area (Figure 1B). Taken together, it seems that both hepatic/liver and thyroid immortalized non-tumorigenic cells at the interphase stage are characterized by the formation of cytoplasmic “DROSHA-bodies” in close asymmetric proximity to a immortalized cell nucleus.

### 2.2. DGCR8-Independent Role(s) of DROSHA Protein in the Cytoplasm of Immortalized Cells at the Interphase Stage

**Figure 2:**
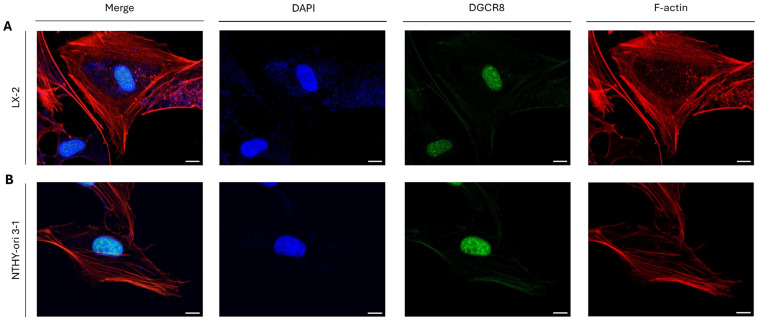
**DGCR8’s sub-cellular distribution in human immortalized non-tumorigenic cells.** CLSM immunofluorescence images of (**A**) LX-2 (human stellate hepatic/liver) and (**B**) NTHY-ori 3-1 (human thyroid follicular/epithelial) cells, revealing DGCR8’s distribution (exclusively in the nucleus). DGCR8 protein is visualized in green, F-actin (F: Filamentous) is shown in red and the cell nucleus is depicted in blue (DAPI). Scale bar: 10 μm.

Since DGCR8 is an essential and indispensable co-factor to the proper action(s) of DROSHA in the nucleus, as the microprocessor complex is capable of pri-miRNA processing only in its entirety and completely assembled structure (Supplementary Figure S1), we next considered if DGCR8 is localized at “DROSHA-bodies” as well. Intriguingly, both in LX-2 (hepatic/liver) (Figure 2A) and NTHY-ori 3-1 (thyroid) (Figure 2B) immortalized cells, the DGCR8 protein presented a strong nuclear topology, although the total absence of its (DGCR8) immunofluorescence-mediated detection in the cytoplasm of the herein examined interphase cells (i.e., LX-2 and NTHY-ori 3-1) could be unambiguously recognized (Figure 2A,Β). Altogether, it seems that “DROSHA-bodies” play microprocessor-independent roles in the cytoplasm of immortalized cells (e.g., LX-2 and NTHY-ori 3-1) during the interphase stage, as strongly suggested by the lack of DGCR8 protein from “DROSHA-bodies”, thereby informing a hitherto unexplored function of DROSHA unrelated to miRNA biogenesis in the cytoplasm of immortalized cells at the interphase stage.

### 2.3. DROSHA Compartmentalization in the Cytoplasm of Liver and Thyroid Cancer Cells During Interphase

**Figure 3:**
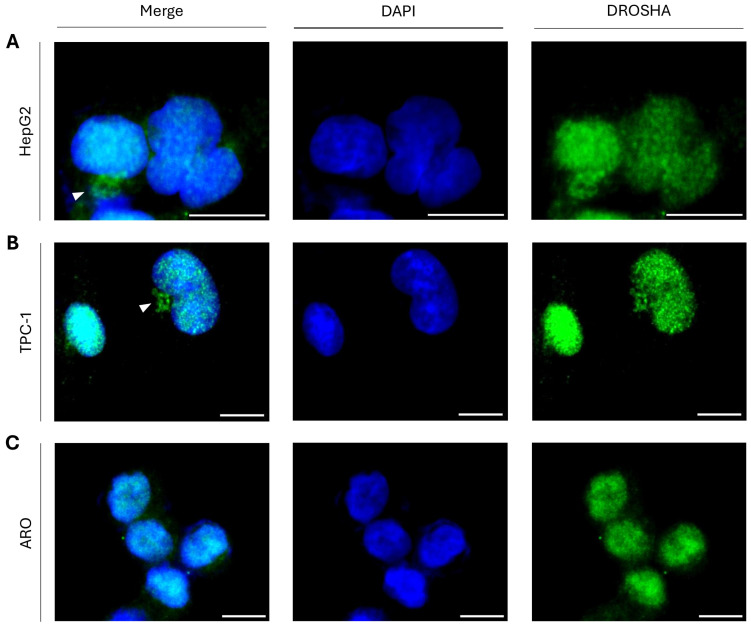
**Sub-cellular distribution of DROSHA protein in human hepatic/liver and thyroid cancer cells.** CLSM immunofluorescence images of (**A**) HepG2 (human hepatocellular carcinoma/liver cancer), (**B**) TPC-1 (human papillary/thyroid carcinoma) and (**C**) ARO (human thyroid/anaplastic carcinoma) cells, presenting DROSHA’s sub-cellular compartmentalization (white arrowheads: “DROSHA bodies”). DROSHA is visualized in green and the cell nucleus is shown in blue (DAPI). Scale bar: 10 μm.

To prove that cytoplasmic DROSHA is not exclusively detected in immortalized non-tumorigenic cells, which usually serve as “normal/physiological” “counterparts” of cancer cells in a given tissue, we next examined DROSHA’s distribution in liver and thyroid cancer cell lines using HepG2 human hepatocellular carcinoma cells as the liver cancer model and TPC-1 human papillary/thyroid carcinoma cells as the thyroid cancer model. In both HepG2 (Figure 3A) and TPC-1 (Figure 3B) interphase cells, we can clearly distinguish the cytoplasmic accumulation of DROSHA protein in close asymmetric proximity to the cell nucleus (Figure 3A,B), thus demonstrating that besides liver and thyroid immortalized non-tumorigenic cells, their tumorigenic “counterparts” (e.g., HepG2 and TPC-1 cancer cells) also present the immunofluorescence-derived phenotype of DROSHA localization in specific cytoplasmic assemblies. However, the ARO human thyroid/anaplastic carcinoma cells seem to lack similar to TPC-1, respective, patterns, with only a few small (in size) cytoplasmic foci being recognized close to some cell nuclei (Figure 3C), thereby indicating the mechanistic association of oncogenic signatures with DROSHA-specific cytoplasmic patterning in human thyroid cancer.

### 2.4. Formation of “DROSHA-Bodies” in the Cytoplasm of Human Urothelial Bladder Cancer Cells: A Phenotypic Universality Unmasking

**Figure 4:**
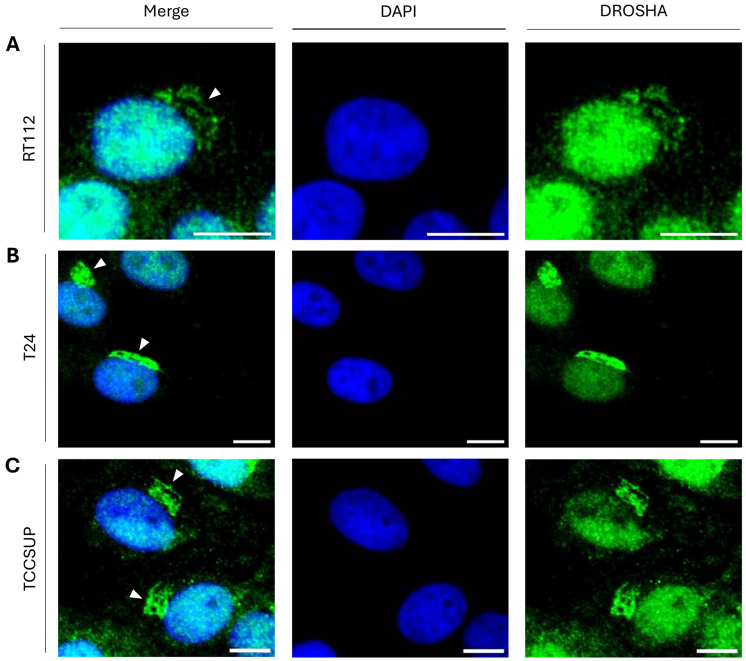
**Patterning of DROSHA’s sub-cellular localization in human urothelial bladder cancer cells of diverse malignancy grades.** CLSM immunofluorescence images of (**A**) RT112 (malignancy grade I/II), (**B**) T24 (malignancy grade III) and (**C**) TCCSUP (malignancy grade IV) cells, showing DROSHA’s distribution in human urothelial bladder cancer cell environments (white arrowheads: “DROSHA bodies”). DROSHA is visualized in green and the cell nucleus is indicated in blue (DAPI). Scale bar: 10 μm.

To further expand the collection of tumor cells that can be profiled by the immunofluorescence-facilitated detection of DROSHA protein in distinct cytoplasmic assemblies, we also investigated DROSHA’s distribution in the three human urothelial bladder cancer cell lines RT112 (malignancy grade I/II; Figure 4A), T24 (malignancy grade III; Figure 4B) and TCCSUP (malignancy grade IV; Figure 4C). Importantly, all three bladder cancer cell lines, herein examined, were being presented with distinct cytoplasmic “DROSHA-bodies” during the interphase stage (Figure 4A–C), thereby indicating the cellular universality of our novel phenotype.

### 2.5. Cytoplasmic Accumulation of DROSHA Does Not Require the p53 Protein in Colon Cancer Cells

**Figure 5:**
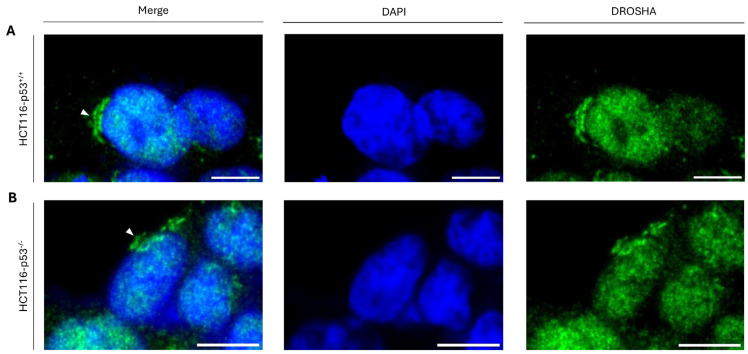
**Profiling of DROSHA’s sub-cellular compartmentalization in human colon cancer cells in the presence or absence of p53 protein.** CLSM immunofluorescence images of (**A**) HCT116-p53^+/+^ and (**B**) HCT116-p53^−/−^ cells, indicating DROSHA’s distribution (white arrowheads: “DROSHA bodies”) in human colon cancer cell settings either retaining (**A**) or lacking (**B**) the p53 protein, respectively. DROSHA is visualized in green and the cell nucleus is pointed out in blue (DAPI). Scale bar: 10 μm.

Considering that T24 cells carry a loss-of-function mutation in the *TP53* gene locus that results in a functionally inactive protein product (p53^ΔΥ126^) [36], we next reasoned that the tumor-suppressor protein p53 may not play an essential role in the cytoplasmic compartmentalization of DROSHA protein. To further investigate this mechanistic uncoupling, we next used the human colon cancer cell line HCT116-p53^−/−^, which lacks a functional p53, to check DROSHA’s distribution, with the parental HCT116-p53^+/+^ counterpart colon cancer cells that express the wild-type p53 protein being engaged as a suitable control. Notably, in both HCT116-p53^+/+^ (Figure 5A) and HCT116-p53^−/−^ (Figure 5B) isogenic cell lines, “DROSHA-bodies” could be easily recognized (besides the nucleus) in the cytoplasm (proximal to the interphase nucleus) (Figure 5A,B), strongly suggesting the non-essential or redundant role(s) of p53 protein in the control of DROSHA’s cytoplasmic localization in colon cancer cell environments.

### 2.6. Metastasis-Independent Assembly of Cytoplasmic “DROSHA-Bodies” in Human Melanoma Cells

**Figure 6:**
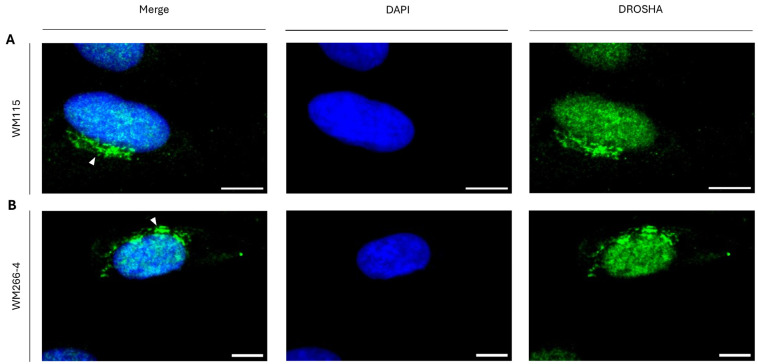
**Sub-cellular compartmentalization of DROSHA protein in human melanoma cells of distinct metastatic dynamics.** CLSM-captured immunofluorescence images of (**A**) WM115 (pre-metastatic) and (**B**) WM266-4 (metastatic) cells (with both cell lines having been derived from the same patient), presenting DROSHA’s distribution (white arrowheads: “DROSHA bodies”). DROSHA is visualized in green and the cell nucleus is shown in blue (DAPI). Scale bar: 10 μm.

Since we have previously found that T24 cells, besides typical tumor xenografts, can induce metastatic incidents in SCID (severe combined immunodeficient) mouse settings, we, subsequently, analyzed DROSHA’s compartmentalization profiles in WM115 (pre-metastatic) and WM266-4 (metastatic) human melanoma cells, both having originated from the same patient. Interestingly, both WM115 (Figure 6A) and WM266-4 (Figure 6B) cell-line populations (both being simultaneously grown and cultured in identical conditions that ensured the absence of stress), at their interphase stage, were characterized by similar to each other DROSHA-specific immunophenotypes, with “DROSHA-bodies” being clearly observed in cytoplasmic areas (besides the typical nuclear ones), asymmetrically close to the respective nuclei (Figure 6A,B), thus demonstrating the metastasis-independent formation of cytoplasmic “DROSHA-bodies” in human melanoma cell settings.

### 2.7. DROSHA Compartmentalization at the Golgi Apparatus in Human Immortalized and Cancer Cells During Interphase

**Figure 7:**
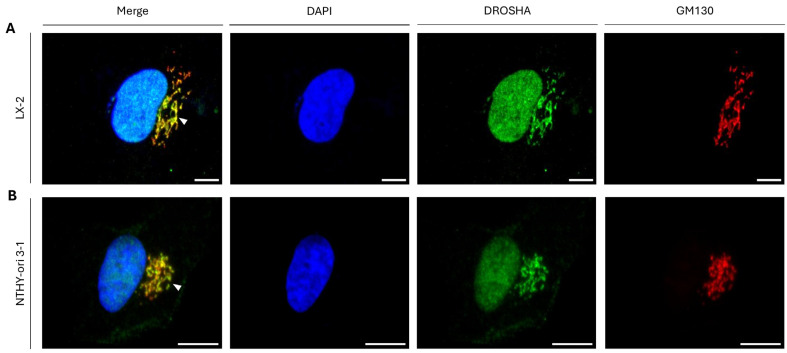
**DROSHA co-localization with the GM130 protein at the Golgi apparatus in human immortalized non-tumorigenic cells.** CLSM-captured immunofluorescence images of (**A**) LX-2 (human stellate hepatic/liver) and (**B**) NTHY-ori 3-1 (human thyroid follicular-epithelial) cells, demonstrating the remarkable co-localization (merger of green and red colors) of DROSHA protein with the Golgi-matrix protein GM130 (white arrowheads: “DROSHA-at-Golgi” immunophenotype). DROSHA is visualized in green, GM130 is shown in red and the cell nucleus is presented in blue (DAPI). Scale bar: 10 μm.

To mechanistically illuminate the nature of “DROSHA-bodies” in the cytoplasm of interphase cells and taking into full consideration the “cisternae”-like morphological/structural patterning of the obtained immunophenotypes, we next examined if “DROSHA-bodies” are specifically compartmentalized at the Golgi apparatus, which is mainly typified by a series of flattened, stacked pouches, typically called “cisternae”. Strikingly, the strong co-immunolocalization profiling being observed in between DROSHA and GM130, a major Golgi-matrix protein [37,38], in both LX-2 and NTHY-ori 3-1 cells (Figure 7A,B), undoubtedly demonstrates the specific compartmentalization of DROSHA protein at the Golgi apparatus of human immortalized non-tumorigenic liver (e.g., LX-2; Figure 7A) and thyroid (e.g., NTHY-ori 3-1; Figure 7B) cells during the interphase stage.

Next, to experimentally strengthen the validity of DROSHA’s capacity to reside at the Golgi organelle, LX-2 cells were transiently transfected with the “CellLight™ Golgi-RFP” construct, which expresses the Red Fluorescent Protein (RFP) fused to the human Golgi apparatus-residing enzyme N-Acetyl-Galactos-Aminyl-Transferase and, subsequently, is processed for immunofluorescence after incubation with the herein used anti-DROSHA primary antibody. The highly overlapping (merger) signal obtained (Supplementary Figure S2) unambiguously proves the specific compartmentalization of “DROSHA-bodies” at the Golgi apparatus in LX-2 interphase cells.

Other than LX-2, both HepG2 (human hepatocellular carcinoma/liver cancer) (Supplementary Figure S3A) and HCT116-p53^+/+^ (human colon cancer; wild-type *TP53*) (Supplementary Figure S3B) cells were also characterized by strong overlaps of the respective immunolocalization patterns in between DROSHA and GM130 proteins, thereby further corroborating the non-canonical compartmentalization of DROSHA protein at the Golgi apparatus in human cancer cells of diverse tissue origin (e.g., liver and colon). Of note, DGCR8 (exclusively in the nucleus) proved unable to co-localize with GM130 (exclusively at the Golgi organelle) in LX-2 (human stellate hepatic/liver) (immortalized) cells (Supplementary Figure S4), thus undoubtedly ensuring the inability of DGCR8 protein to compartmentalize at the Golgi system in human immortalized (hepatic/liver) cells.

Altogether, it seems that in contrast to DGCR8, its major interactor (in the nucleus), DROSHA, can be non-canonically compartmentalized with GM130 at the Golgi apparatus, most likely providing miRNA biogenesis-independent activity(ties) in a spatially specific fashion.

### 2.8. Immunophenotypic Variations of Golgi Apparatus-Containing DROSHA Protein in Human Immortalized Cells

**Figure 8:**
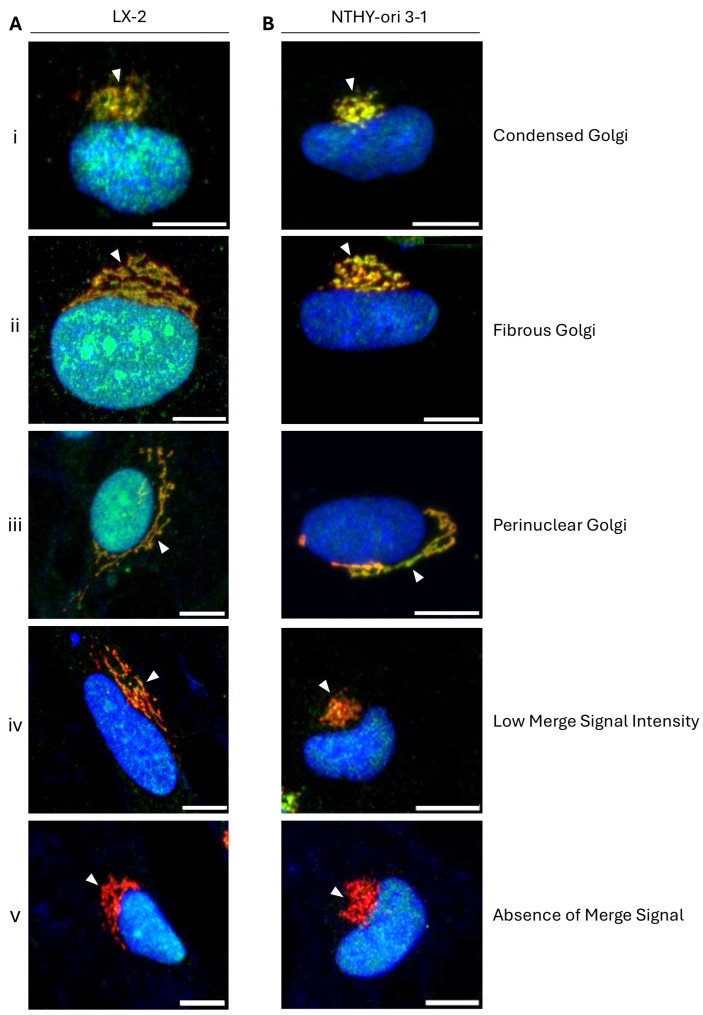
Immunophenotypic patterning variety of Golgi apparatus-containing DROSHA structural architecture in human immortalized non-tumorigenic cells. CLSM-mediated immunofluorescence images of (**A**) LX-2 (human stellate hepatic/liver) and (**B**) NTHY-ori 3-1 (human thyroid follicular/epithelial) cells, describing the variety (**i**–**v**) in the architectural organization of Golgi apparatus-containing DROSHA and the multiplicity (**i**–**v**) of DROSHA and GM130 co-localization (merger of green and red colors) patterns at the Golgi system (white arrowheads: “DROSHA-at-Golgi” immunophenotypes). DROSHA is visualized in green, GM130 is presented in red and the cell nucleus is shown in blue (DAPI). Scale bar: 10 μm.

During extensive cell examination, various configurations of the Golgi system-accommodating DROSHA (“DROSHA-at-Golgi”) immunophenotype were observed. Specifically, the “DROSHA-at-Golgi” pattern was identified comparatively as more “condensed” (or “bulky”) (Figure 8i) in some cells and more “fibrous” (or “stacky”) (Figure 8ii) in other cells, and either (asymmetrically) “polarized” (Figure 8i,ii) or, rather, (symmetrically) “perinuclear” (Figure 8iii) in distinct cell groups, with DROSHA protein being strongly compartmentalized at the Golgi organelle regardless of its immunophenotypic formation in hepatic/liver (LX-2) or thyroid (NTHY-ori 3-1) human immortalized cells (Figure 8A,B(i–v)). Nevertheless, despite the strong accumulation of DROSHA protein at the Golgi system in most of the examined cells, in some cases, there was (comparatively) lower (merger) signal intensity (Figure 8iv) or even a complete absence (Figure 8v) of DROSHA immunodetection at the Golgi apparatus.

Altogether, our results inform a possible condition-dependent localization of DROSHA protein at the Golgi organelle, as its compartmentalization fashion could be (local) stress-related and/or cell cycle-regulated without excluding the alternative scenario of different cell sub-populations carrying various oncogenic signatures.

### 2.9. “DROSHA-At-Golgi” Is Being Unveiled as a Novel, Compelling Biomarker for Human Malignancies in an Oncogenic Signature-Specific Manner

**Figure 9:**
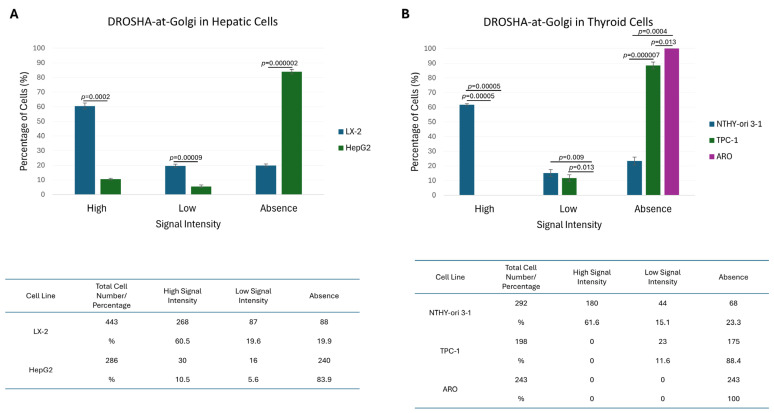
**Comparative quantification of human immortalized non-tumorigenic versus cancer cells containing DROSHA-specific patterns that are tightly associated with the Golgi apparatus.** Histograms (top) and tables (bottom) presenting the total cell number and percentage (%) of cells with high, low and no “DROSHA-at-Golgi” signal intensities in the (**A**) LX-2 (human stellate hepatic/liver) and HepG2 (human hepatocellular carcinoma/liver cancer) and (**B**) NTHY-ori 3-1 (human thyroid follicular/epithelial), TPC-1 (human papillary/thyroid carcinoma) and ARO (human thyroid/anaplastic carcinoma) cell lines using CLSM-derived immunofluorescence images. All the obtained data have derived from three independent biological experiments, and the numerical values are herein presented as “Mean ± Standard Deviation (SD) of the Mean”. Only statistically significant *p* values (*p* < 0.05) are indicated at the top of each respective bar. A statistical analysis was conducted through engagement of the IBM SPSS (v. 26) statistical package, with the significance of the obtained results evaluated using “Two-tailed” Student’s *t*-test.

**Figure 10:**
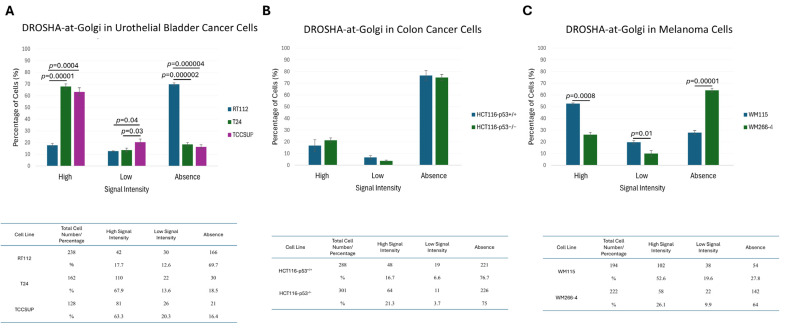
**Quantification of “DROSHA-at-Golgi” immunophenotypes in human cancer cells of diverse malignancy-grade, *TP53* gene-mutation profile and metastatic capacity.** Histograms (**top**) and tables (**bottom**) describing the total number and the (respective) percentage (%) of human cancer cells characterized by high, low and no “DROSHA-at-Golgi” signal intensities in the (**A**) RT112 (human urothelial bladder cancer; malignancy grade I/II), T24 (human urothelial bladder cancer; malignancy grade III) and TCCSUP (human urothelial bladder cancer; malignancy grade IV) cells; (**B**) HCT116-p53^+/+^ (human colon cancer; wild-type *TP53*) and HCT116-p53^−/−^ (human colon cancer; knocked-out *TP53*) cells; and (**C**) WM115 (human melanoma; pre-metastatic) and WM266-4 (human melanoma; metastatic) (WM115 and WM266-4 have originated from the same patient) cell lines via engagement of CLSM-derived immunofluorescence images. All the obtained data were derived from three independent biological experiments, and the numerical values are herein presented as “Mean ± Standard Deviation (SD) of the Mean”. Only statistically significant *p* values (*p* < 0.05) are indicated at the top of each respective bar. Statistical analysis was conducted through engagement of the IBM SPSS (v. 26) statistical package, with the significance of the obtained results evaluated using “Two-tailed” Student’s *t*-test.

Given the identification of cells presenting (comparatively) lower signal intensity or a complete lack of Golgi apparatus-associated DROSHA immunophenotype, together with the absence of the “DROSHA-at-Golgi” axis in ARO (thyroid cancer) cells, we next considered if “DROSHA-at-Golgi” could serve as an effective biomarker for human malignancies. Hence, we measured the percentage of cells either containing or missing the “DROSHA-at-Golgi” phenotype for each cell line, herein examined, and suitably compared the quantified results. Surprisingly, both hepatic/liver (HepG2) (Figure 9A) and thyroid (TPC-1 and ARO) (Figure 9B) cancer cells were presented with significantly higher percentages of the “DROSHA-at-Golgi” missing pattern (i.e., “absence”) compared to immortalized non-tumorigenic cell (LX-2 and NTHY-ori 3-1) respective ones (Figure 9A,B). Intriguingly, in human urothelial bladder cancer cell settings, an opposite association was revealed, with low malignancy-grade cells (RT112: I/II) exhibiting an increased “DROSHA-at-Golgi”-axis value compared to the high(er) malignancy-grade cell (T24: III and TCCSUP: IV), respective ones (Figure 10A)—an unexpected reverse association that may be mechanistically related to the mutational burden and/or developmental origin of the urothelial bladder cancer cells herein examined.

Most importantly, p53 protein does not seem to control DROSHA’s distribution at the Golgi system, as the respective percentages of the “DROSHA-at-Golgi” phenotype in between HCT116-p53^+/+^ and HCT116-p53^−/−^ isogenic (human) colon cancer cell lines did not significantly differ from each other (Figure 10B). However, a notably decreased percentage of “DROSHA-at-Golgi”-positive cells could be (equally) measured for both cell types, thus further corroborating the general association between advanced malignancy-grade profiling and reduced “DROSHA-at-Golgi” immunophenotypic patterning.

In contrast to p53, metastatic traits seem to be critically coupled with diminished “DROSHA-at-Golgi” phenotypic features, as clearly indicated from their respective significant differences recognized between pre-metastatic (WM115) and metastatic (WM266-4) human melanoma cells (Figure 10C). It may be the metastatic micro-environment that can prevent the translocation of DROSHA protein to Golgi apparatus, thereby dictating the essential contribution of the “DROSHA-at-Golgi” axis in negatively controlling the invasion and metastasis of human melanoma cells.

Taken together, we herein unveil an emerging role of DROSHA protein in the initiation and progression of human malignancy. The strong correlation of the “DROSHA-at-Golgi” axis with a cancer cell-specific phenotype, malignancy grade and metastatic profile could suggest “DROSHA-at-Golgi” as a new potential biomarker and therapeutic target for diverse types of human cancer.

### 2.10. DROSHA/GM130 Protein/Protein Interaction (PPI): A Molecular Mechanism for DROSHA’s Compartmentalization at the Golgi Apparatus

**Figure 11:**
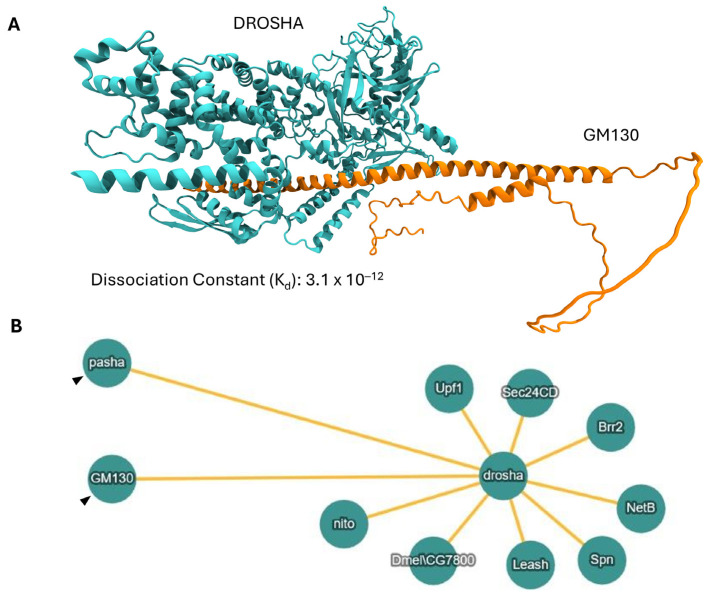
**DROSHA protein-specific bi-molecular interactions.** (**A**) Three-dimensional (3D) molecular model resulting from docking test between DROSHA (cyan color) and GM130 (orange color), a major Golgi-matrix protein. The obtained Dissociation Constant (DC) value (K_d_) indicates the possibility (complex stability) of bi-molecular binding between the two human proteins. (**B**) Schematic illustration of bi-molecular interactions of DROSHA/drosha protein in *Drosophila melanogaster* “interactome”, based on “IntAct Molecular Interaction Database”. Protein/protein interactions (PPIs) of DROSHA/drosha with pasha (DGCR8 in humans) and (*Dm*)GM130 proteins are distinguished (black arrowheads).

Given the strong co-localization profiles of DROSHA and GM130 proteins at the Golgi system of the herein examined cell lines, we next questioned if these two molecules could interact physically and directly, leading to DROSHA’s recruitment and retention at the Golgi apparatus. Hence, we used an “*in silico*” approach of advanced molecular modeling and, specifically, performed protein complex predictions of DROSHA and GM130 structurally modeled proteins. Remarkably, the results demonstrated a potentially strong binding between DROSHA (accession number: Q9NRR4; retrieved from UniProt) and GM130 (accession number: Q5PXD5; retrieved from UniProt) according to the Dissociation Constant (DC) value (K_d_: 3.1 × 10^−12^) of the putative complex. This supports our experimental observations since such a low K_d_ value suggests an intrinsic structural stability of the bi-molecular complex (DROSHA-GM130) (Figure 11A).

Furthermore, “*in silico*” analyzing the composition of DROSHA’s interactome in *Drosophila melanogaster* [39], we strikingly pinpointed (among others) the (*Dm*)GM130 protein as a major (*Dm*)DROSHA/drosha interactor (Figure 11B), clearly demonstrating that DROSHA and GM130 can form stable and functional dimers via physical and direct interaction both “*in silico*” and “*in vivo*”. Our results show that the two proteins most likely interact physically and directly to each other, and it is this structurally stable interaction that can serve as a critical molecular mechanism for the recruitment and retention of DROSHA protein at the Golgi apparatus.

### 2.11. Impact of DROSHA Gene Alterations in Diverse Human Malignancies

**Figure 12:**
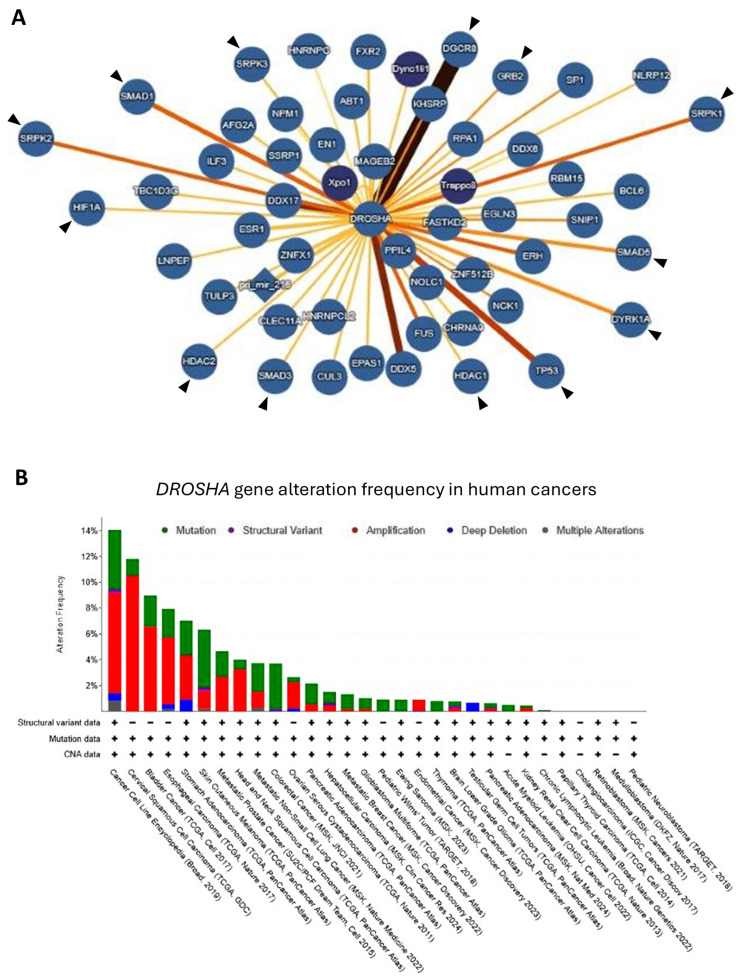
**DROSHA protein/protein interactions (PPIs) and *DROSHA* gene aberrations in human cancers.** (**A**) Interaction network of DROSHA protein in humans based on “IntAct Molecular Interaction Database”. The (comparatively) strongest bi-molecular interactions of DROSHA protein are distinguished with thicker connection lines (e.g., DROSHA and DGCR8), while black arrowheads indicate critical DROSHA interactors, carrying major signaling contribution to oncogenic-network activities. (**B**) Histogram showing the *DROSHA* gene-alteration frequency in multiple human malignancies (cBioPortal Database; cBioPortal for Cancer Genomics, https://www.cbioportal.org/, 2025).

To evolutionarily expand the (*Dm*)DROSHA/drosha molecular interactome, we next examined the DROSHA-specific interaction network in *Homo sapiens*. Although no known interaction with GM130 has been hitherto recognized, thus justifying the absence of the (*Hs*)GM130 interactor from the human collection, multiple other interactors can be mapped, with DGCR8 presenting, as expected, the strongest interaction capacity (the thicker the connection line, the stronger the interaction) among all (Figure 12A). The *H. sapiens* interaction map of DROSHA protein seems to contain, among others, components that are critically involved in the cell cycle control (e.g., SMADs, HDAC2 and GRB2) and/or tumorigenesis (e.g., p53, SRPKs and DYRK1A), presumably fostering their Golgi apparatus-specific compartmentalization and new locality-dependent functionality during carcinogenesis.

In this context, we next examined the impact of *DROSHA* gene alterations in diverse human malignancies. Importantly, high percentages of *DROSHA* mutations can be identified in a wide range of human cancers, with gene amplification being recognized as the most frequent aberration in most of the cases herein studied (Figure 12B) (cBioPortal Database; cBioPortal for Cancer Genomics, https://www.cbioportal.org/, 2025). Indeed, *DROSHA* copy-number variation seems to serve as a critical mechanism of miRNA regulation and homeostasis in cancer, thus indicating an oncogenic role for DROSHA protein, as previously reported [40]. Whether “nuclear” DROSHA (canonical pattern) differs from “Golgi” DROSHA (non-canonical pattern) in molecular activity and oncogenic capacity remains an open issue that needs to be further investigated.

## 3. Discussion

Pri-miRNA to pre-miRNA processing is the main and best-known function of DROSHA protein (Supplementary Figure S1). However, our results indicate new activities of DROSHA in the cytoplasm and, specifically, at the Golgi apparatus, which are most likely miRNA-independent, as pointed out from the absence of its major co-factor/interactor, the DGCR8 protein. Importantly, neither DICER nor AGO2, two fundamental components of the RNAi machinery, can be immunodetected in “DROSHA-bodies” at the Golgi organelle (Supplementary Figure S5), further corroborating the miRNA/RNAi-independent character of “DROSHA-at-Golgi” axis. It may be DROSHA’s new putative properties to critically controlling major cytoplasmic processes that are being mechanistically coupled with the Golgi organelle, such as Golgi integrity, Golgi signaling and Golgi response to stress [41,42,43,44].

Deeply and thoroughly expanding previous observations [45], we herein demonstrate the specific compartmentalization of DROSHA at the Golgi apparatus in a number of human immortalized non-tumorigenic and cancer cell lines of diverse tissue origin, mutational load, malignancy grade and metastatic potential. It is the Golgi apparatus—and not the Endoplasmic Reticulum (ER) (Supplementary Figure S6)—that enables DROSHA’s recruitment and localization at the organelle, with the Golgi system’s environment productively favoring DROSHA targeting and functionality in Golgi organelle stacks and ribbons. The identification of this novel immunophenotype in human cells of diverse tumorigenicity and developmental origin suggests the putatively essential contribution of DROSHA protein to Golgi’s structural integrity, molecular signaling and/or response to stress.

Since the Golgi-matrix protein GM130 has recently proved capable of recruiting RNA molecules and also associating with RNA-binding proteins at the Golgi system membrane, the compartmentalization of DROSHA protein at the Golgi apparatus emerges as a mechanistically compelling issue that necessitates further illumination and molecular dissection. It seems that RNAs can act as structural biopolymers that, together with GM130, ensure Golgi system’s structural maintenance with RNA dissociation under stress conditions, disrupting the integrity and function of the Golgi organelle [37]. Hence, given the dual capacity of DROSHA to physically interact with GM130 (Figure 11) and to also bind to double-stranded RNA (dsRNA) species (Supplementary Figure S1), we herein suggest a new molecular property of DROSHA to serve as a putative protein “linker/bridge” between GM130 and (small) (ds)RNA entities, all being organized in multiple trimeric complexes and specifically compartmentalized at the Golgi apparatus, ultimately providing the required structural stability and functional flexibility of both stacks and ribbons of the organelle during cell survival, growth, division, migration, differentiation and stress response. Of note, as GM130 is significantly involved in cell cycle progression, cell polarization, (directed) cell migration and the disassembly/reassembly of the Golgi system during mitosis [38], the herein observed immunophenotypic variations of DROSHA’s architectural organization in human cells (Figure 8) most likely reflect the cardinal role(s) of DROSHA protein in the control of Golgi apparatus structural alterations required to occur during cell proliferation and migration processes.

In conclusion, the strong immunodetection pattern of DROSHA protein at the Golgi system, together with the lack of DGCR8, DICER and AGO2—all major components of the miRNA/RNAi machinery—at the Golgi apparatus constitute the most remarkable finding of the present study, which strongly suggests that DROSHA protein can exert miRNA/RNAi-independent activity(ties) at the Golgi apparatus environment via the engagement of novel mechanisms that need to be further illuminated and functionally dissected. The herein demonstrated novel miRNA/RNAi-independent function(s) of the “DROSHA-at-Golgi” axis can undoubtedly support its prompt engagement in differential diagnosis and clinical management of human cancer. Its immunophenotypic variations that seem to depend on molecular signature (e.g., tissue origin), oncogenic activity (e.g., malignancy grade/mutational burden) and metastatic profile (e.g., low versus high) render “DROSHA-at-Golgi” a promising diagnostic and prognostic biomarker in cancer, warranting further validation in clinical cohorts.

## 4. Materials and Methods

### 4.1. Cell Lines—Cell Cultures

LX-2 (human stellate hepatic/liver, immortalized non-tumorigenic, cells) (Sigma-Aldrich, St. Louis, MO, USA), HepG2 (human hepatocellular carcinoma/liver cancer-derived cells) (ATCC-LGC Standards, Oxford, UK), RT112 (human urothelial bladder-cancer-derived cells; malignancy grade I/II;kindly provided by Professor John R. Masters, London, UK), T24 (human urothelial bladder-cancer-derived cells; malignancy grade III) (ATCC-LGC Standards GmbH, Wesel, Germany), TCCSUP (human urothelial bladder-cancer-derived cells; malignancy grade IV) (ATCC-LGC Standards GmbH, Wesel, Germany), HCT116-p53^+/+^ (human colon cancer-derived cells; wild-type *TP53*; kindly provided by Professor Jean-Christophe Marine, Leuven, Belgium), HCT116-p53^−/−^ (human colon cancer-derived cells; knocked-out *TP53*; kindly provided by Professor Jean-Christophe Marine, Leuven, Belgium), WM115 (human melanoma-derived cells; pre-metastatic) (ECACC/Sigma-Aldrich, Munich, Germany) and WM266-4 (human melanoma-derived cells; metastatic) (ECACC/Sigma-Aldrich, Munich, Germany; WM115 and WM266-4 cells were derived from the same patient) cell lines were cultured in 1x DMEM growth medium (41966-029; Gibco, Thermo Fisher Scientific, Waltham, MA, USA), while NTHY-ori 3-1 (human thyroid follicular-epithelial, immortalized non-tumorigenic, cells; Sigma-Aldrich, St. Louis, MO, USA), TPC-1 (human papillary/thyroid carcinoma-derived cells; Sigma-Aldrich, St. Louis, MO, USA) and ARO (human thyroid-anaplastic carcinoma-derived cells; ATCC, Manassas, VA, USA) cell lines were cultured in 1x RPMI-1640 growth medium (61870-010; Gibco, Thermo Fisher Scientific, MA, USA) in standard conditions (37 °C and 5% CO_2_). Each medium was supplemented with 10% Fetal Bovine Serum (FBS) (16000044; Gibco, Thermo Fisher Scientific, MA, USA), 1% Penicillin/Streptomycin (10378016; Gibco, Thermo Fisher Scientific, MA, USA) and 1% L-Glutamine (BEBP17-605E; Lonza, Verviers, Belgium).

### 4.2. Transfection

LX-2 (human stellate hepatic/liver, immortalized non-tumorigenic) cells were transiently transfected at their early “culture-splitting” passages (e.g., third splitting stage), 24 h post-seeding, with the expression plasmid “CellLight™ Golgi-RFP” (C1059-3; Invitrogen, Thermo Fisher Scientific, MA, USA), whose cognate protein product serves as a Golgi apparatus-specific marker using Lipofectamine 2000 (Invitrogen, Thermo Fisher Scientific, MA, USA) according to the manufacturer’s instructions. Cells were collected 16 h post-transfection and immediately processed for immunofluorescence (Lipofectamine 2000-only treated cells served as negative control). All transfection experiments were repeated three independent times.

### 4.3. Immunofluorescence

Cells at their early “culture-splitting” passages (e.g., third splitting stage) were cultured on 10 mm round coverslips; 24 h post-seeding, they were then fixed in 4% paraformaldehyde (P6148; Sigma-Aldrich, St. Louis, MO, USA) solution for 10 min at room temperature (RT). Then, cells were membrane-permeabilized by their incubation in 0.1% Triton-X 100 (1086031000, Sigma-Aldrich, St. Louis, MO, USA), followed by blocking with 5% BSA (A7906, Bovine Serum Albumin; Merck KGaA, Darmstadt, Germany) for 1 h at RT. Subsequently, cells were exposed to the specific anti-DROSHA (ab12286; Abcam, Cambridge, UK; 1:100), anti-DGCR8 (NBP1-30115; Novus Biologicals, Centennial, CO, USA; 1:100), anti-GM130 (ab169276; Abcam, Cambridge, UK; 1:100), anti-AGO2 (ab57113; Abcam, Cambridge, UK; 1:200) and anti-DICER (NBP1-06520; Novus Biologicals, Centennial, CO, USA; 1:100) primary antibodies for 16 h at 4 °C. Next, cells were exposed to the Goat anti-Mouse IgG (H+L), Alexa Fluor™ 568 (A-11004; Invitrogen, Thermo Fisher Scientific, MA, USA; 1:500) and Donkey anti-Rabbit IgG (H+L), Alexa Fluor™ 488 (A-21206; Invitrogen, Thermo Fisher Scientific, MA, USA; 1:500) secondary antibodies for 1 h at RT. For F-Actin (F: Filamentous) visualization, cells were incubated with Rhodamine Phalloidin (R415; Invitrogen, Thermo Fisher Scientific, MA, USA; 1:400) for 30 min at RT. Vectashield^®^ Mounting Medium with DAPI (H-1200, Vector Laboratories Inc., Newark, CA, USA) was used to visualize cell nuclei at a 405 nm excitation wavelength. Secondary antibody-only treated cells served as the negative control for each condition herein tested. All immunofluorescence experiments were repeated three independent times.

### 4.4. Confocal Laser Scanning Microscopy

Observation of immunofluorescent and fluorescent cells was carried out using a Confocal Laser Scanning Microscope (CLSM) Leica SP5 (Leica Microsystems GmbH, Wetzlar, Germany) and LAS-AF (version 2.7.3.9723) software (Leica Microsystems GmbH, Wetzlar, Germany) with (a) 63× objective magnification; (b) 1024 × 1024 pixel dwell time; (c) 561 (red), 488 (green) and 405 nm (blue) laser wavelengths; and (d) 0.5 μm Z-stack step (followed by Z-projection). For Z-projection, color-splitting, color-merging and scale-bar determination, the ImageJ (version 1.54) open-source software was suitably executed. Twenty non-overlapping CLSM fields for each examined condition (e.g., different cell line) per experiment were used to manually quantify the “DROSHA-at-Golgi” phenotype.

### 4.5. Molecular Modeling

Predictions of DROSHA-GM130 putative complexes were generated by AlphaFold Server [46] with “seed = 42” using protein sequences (accession numbers: Q9NRR4 (DROSHA) and Q5PXD5 (GM130)) retrieved from UniProt [47]. Conventional docking methods could not be applied, as there was no experimental structure for GM130 and DROSHA is a very large protein (1374 amino acids; Ref. [47]). One of the five inferred poses was chosen considering its Dissociation Constant (K_d_), which was predicted by Prodigy Server [48]. The lower the K_d_ value, the stronger the binding affinity. The selected pose was converted to Protein Data Bank (PDB) format via Gemmi [49] Python package (version 0.7.0) for compatibility with PDBsum Server [50], which was utilized for detecting the interfacing residues. The potential complex was visualized via VMD (version 1.9.3) [51].

### 4.6. Morphometric Measurements—Statistical Analysis

All obtained images were thoroughly evaluated, and individual cells (~10,000 single cells) were suitably categorized into three groups based on the absence or presence (e.g., high or low) of the “DROSHA-at-Golgi” phenotype, with each category’s content being manually quantified and graphically described. Data were derived from three independent biological experiments and numerical values and are herein presented as “Mean ± Standard Deviation (SD) of the Mean”, with “*p* values” appropriately and respectively indicated. All statistical analyses were conducted via employment of the IBM SPSS (version 26) statistical package, with the significance of the obtained results being evaluated using a “Two-tailed” Student’s *t*-test.

## 5. Conclusions

Through the employment of advanced cell imaging and structural bioinformatics technologies, we herein demonstrate the non-canonical and strong compartmentalization of DROSHA protein at the Golgi apparatus in a variety of human cancer cell lines. Our findings indicate the miRNA/RNAi-independence—but also Golgi-dependence—of novel DROSHA roles that may be potentially involved in critical oncogenic activities of human cells. “DROSHA-at-Golgi” phenotypic association with malignancy grade and metastatic dynamics seems to support the essential contribution of Golgi-residing DROSHA to cancer cell aggressiveness. *In toto*, our results suggest “DROSHA-at-Golgi” as a promising prognostic/diagnostic biomarker and therapeutic target for human malignancy, although further validation in diverse cancer types and clinical cohorts is warranted. The employment of spatial proteomics for single-cell and organelle-specific profiling [52,53] is expected to increase the resolution power for Golgi apparatus mapping both at the “bench” and “bedside” levels, thereby opening new translational and therapeutic windows for organellar (“Golgian”) DROSHA targeting in health and disease.

## Data Availability

All data are available in the main text or Supplementary Materials.

## Supplementary Materials

The following supporting information can be downloaded at https://www.mdpi.com/article/10.3390/ijms26199319/s1.
