# Supplementary material for: Non-Canonical Compartmentalization of DROSHA Protein at the Golgi Apparatus: miRNA Biogenesis-Independent Functionality in Human Cancer Cells of Diverse Tissue Origin
Source: Int J Mol Sci. 2025 Sep 24;26(19):9319. doi: 10.3390/ijms26199319 (PMC12525405; doi:10.3390/ijms26199319)
Supplement: Supplementary file 1 [file ijms-26-09319-s001.zip › ijms-3873943-supplementary.pdf]

International Journal of
Molecular Sciences

MDPI

Article – Supplementary Figures

Non-Canonical Compartmentalization of DROSHA Protein at the Golgi Apparatus: miRNA Biogenesis-Independent Functionality in Human Cancer Cells of Diverse Tissue Origin

Eleni I. Theotoki [1,2], Panos Kakoulidis [3], Kostas A. Papavassiliou [4], Konstantinos-Stylianos Nikolakopoulos [1], Eleni N. Vlachou [1], Efthimia K. Basdra [5], Athanasios G. Papavassiliou [5], Ourania E. Tsitsilonis [6], Gerassimos E. Voutsinas [7], Athanassios D. Velentzas [1], Ema Anastasiadou [2,†] and Dimitrios J. Stravopodis [1,*]

1 Section of Cell Biology and Biophysics, Department of Biology, School of Science, National and Kapodistrian University of Athens (NKUA), 157 01 Athens, Greece; elthk@biol.uoa.gr (E.I.T.); ksnikolakop@biol.uoa.gr (K.-S.N.); eleni.vlachou.2002@gmail.com (E.N.V.); tveletz@biol.uoa.gr (A.D.V.)

2 Center of Basic Research, Biomedical Research Foundation of the Academy of Athens (BRFAA), 115 27 Athens, Greece; anastasiadou@bioacademy.gr

3 Department of Informatics and Telecommunications, School of Science, National and Kapodistrian University of Athens (NKUA), 157 01 Athens, Greece; pkakoulidis@di.uoa.gr

4 First University Department of Respiratory Medicine, "Sotiria" Chest Hospital, Medical School, National and Kapodistrian University of Athens (NKUA), 115 27 Athens, Greece; konpapav@med.uoa.gr

5 Department of Biological Chemistry, Medical School, National and Kapodistrian University of Athens (NKUA), 115 27 Athens, Greece; ebasdra@med.uoa.gr (E.K.B.); papavas@med.uoa.gr (A.G.P.)

6 Section of Animal and Human Physiology, Department of Biology, School of Science, National and Kapodistrian University of Athens (NKUA), 157 01 Athens, Greece; rtsitsil@biol.uoa.gr

7 Laboratory of Molecular Carcinogenesis and Rare Disease Genetics, Institute of Biosciences and Applications, National Center for Scientific Research (NCSR) "Demokritos", 153 10 Athens, Greece; mvoutsin@bio.demokritos.gr

* Correspondence: dstravop@biol.uoa.gr; Tel.: +30-210-727-4105

† Present Address: Department of Health Science, Higher Colleges of Technology (HCT), Academic City Campus, Dubai 171 55, United Arab Emirates; eanastasiadou@hct.ac.ae

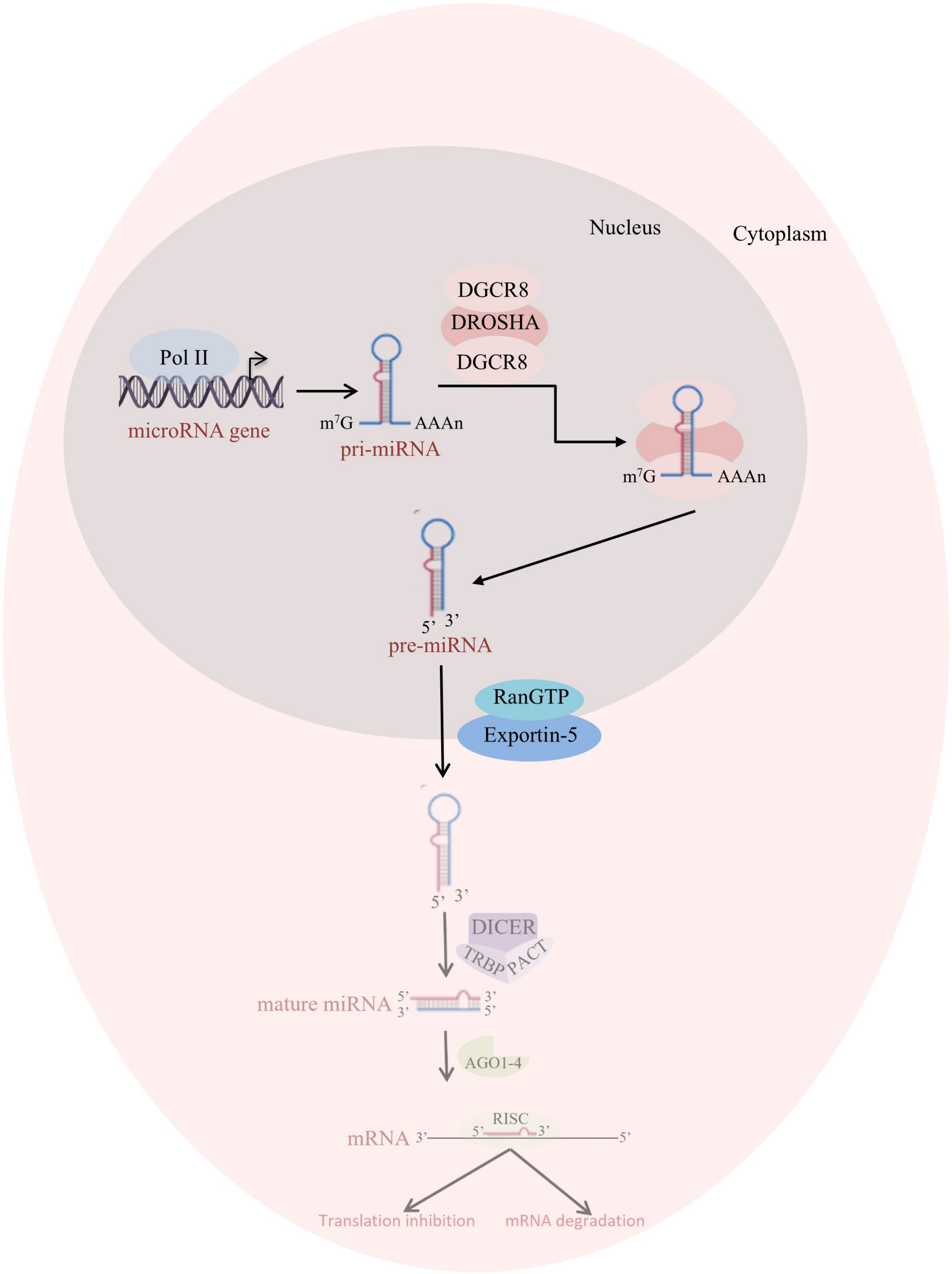

Supplementary Figure S1. Graphic illustration of the major functional role of DROSHA protein in a typical cell nucleus. During the canonical pathway of miRNA biogenesis, the primary miRNAs (pri-miRNAs), transcribed by the RNA Polymerase II (Pol II), are cleaved by the Microprocessor complex DROSHA-DGCR8 (1:2 molecular stoichiometry), generating the precursor miRNAs (pre-miRNAs), which are, then, being exported from the nucleus to the cytoplasm, for further processing, by the "DICER-AGO" system engagement.

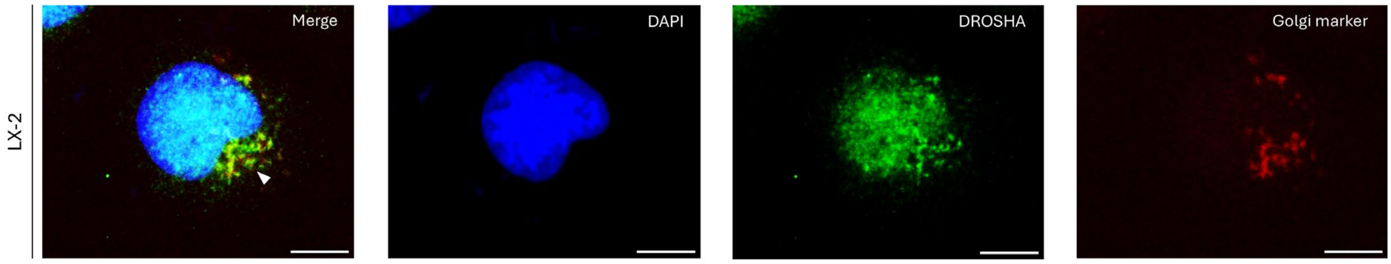

Supplementary Figure S2. DROSHA's compartmentalization at the Golgi apparatus in human hepatic/liver, immortalized/"normal", cells. CLSM-captured immunofluorescence images of LX-2 (human stellate hepatic/liver) cells, presenting highly overlapping signals of endogenous DROSHA protein and (transiently) transfected "CellLight™ Golgi-RFP" plasmid that produces a Golgi system-specific marker (white arrowhead: "DROSHA-at-Golgi" immunophenotype). DROSHA is visualized in green colour; Golgi apparatus-specific genetic marker is shown in red colour; and cell nucleus is presented in blue colour (DAPI). Scale bar: 10 μm.

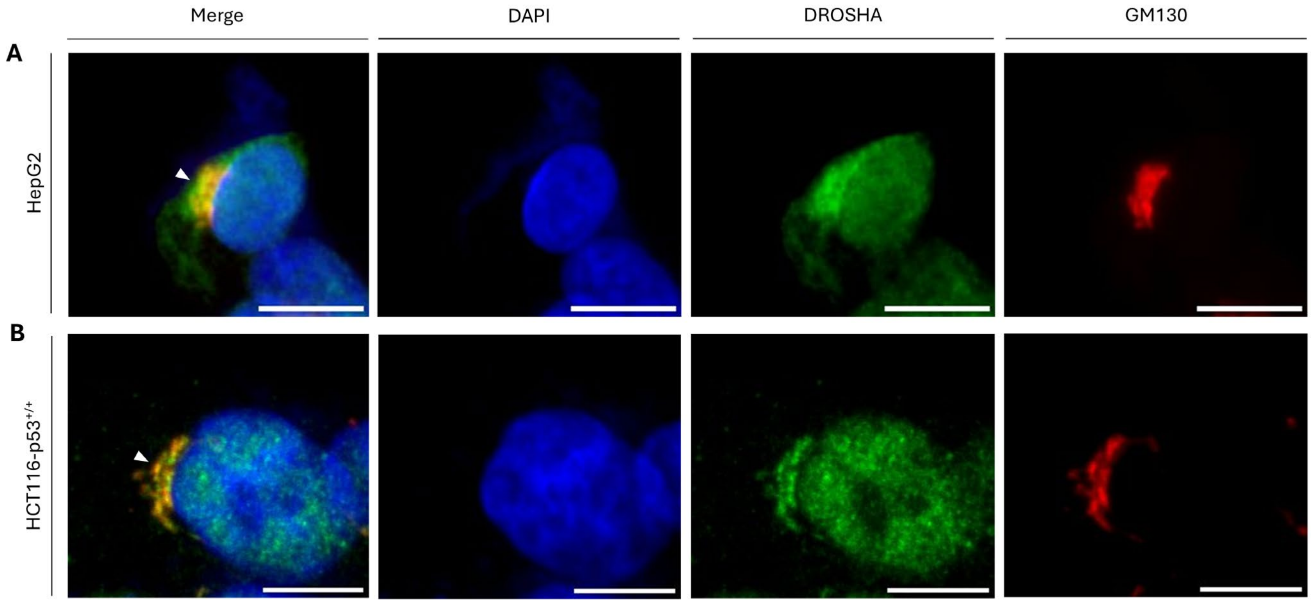

Supplementary Figure S3. Co-compartmentalization of DROSHA and GM130 proteins at the Golgi organelle in human cancer cells. CLSM-captured immunofluorescence images of (**A**) HepG2 (human hepatocellular carcinoma/liver cancer) and (**B**) HCT116-p53$^{+/+}$ (human colon cancer; wild-type *TP53*) cells, presenting highly overlapping immunodetection signals (merger of green and red colours) of DROSHA and GM130 proteins at the Golgi apparatus (white arrowheads: "DROSHA-at-Golgi" immunophenotype). DROSHA is visualized in green colour; GM130 (Golgi apparatus-specific biomarker) is shown in red colour; and cell nucleus is presented in blue colour (DAPI). Scale bar: 10 μm.

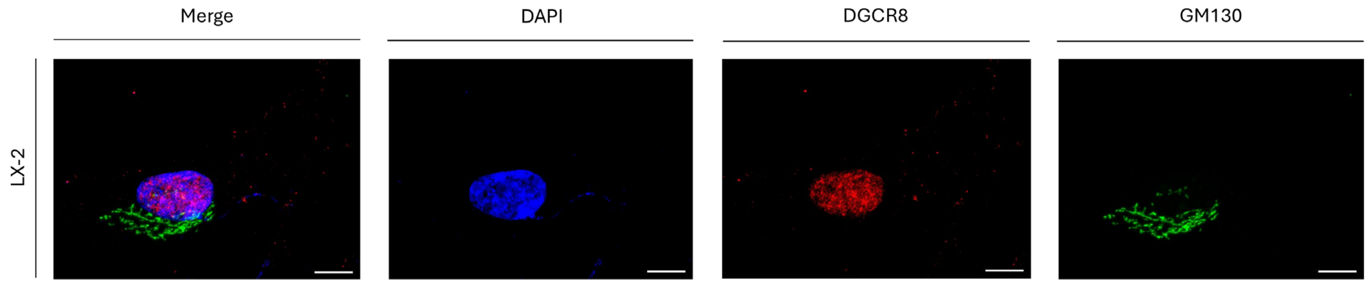

Supplementary Figure S4. Lack of DGCR8 and GM130 co-localization patterning at the Golgi apparatus in LX-2 cells. CLSM-captured immunofluorescence images of LX-2 (human stellate hepatic/liver) cells, demonstrating the absence of overlapping immunodetection signals in between DGCR8 (in the nucleus) and GM130 (at the Golgi system) proteins. DGCR8 is visualized in red colour; GM130 (Golgi organelle-specific biomarker) is shown in green colour; and cell nucleus is presented in blue colour (DAPI). Scale bar: 10 μm.

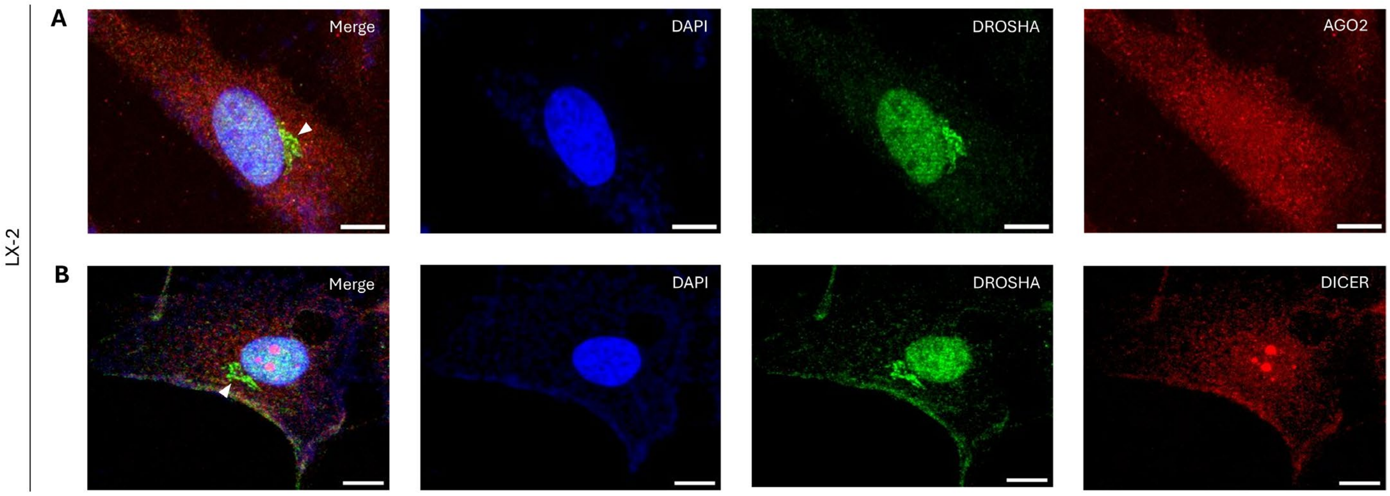

Supplementary Figure S5. Golgi apparatus lacks AGO2 and DICER proteins in human hepatic/liver, immortalized/"normal", cells. CLSM-derived immunofluorescence images of LX-2 (human stellate hepatic/liver) cells, demonstrating the absence of co-localization patterning in between DROSHA and (**A**) AGO2 or (**B**) DICER (fundamental RNAi-machinery components), at the Golgi apparatus (white arrowheads: "DROSHA-at-Golgi" immunophenotype). DROSHA is visualized in green colour; AGO2 and DICER are shown in red colour; and cell nucleus is presented in blue colour (DAPI). Scale bar: 10 μm.

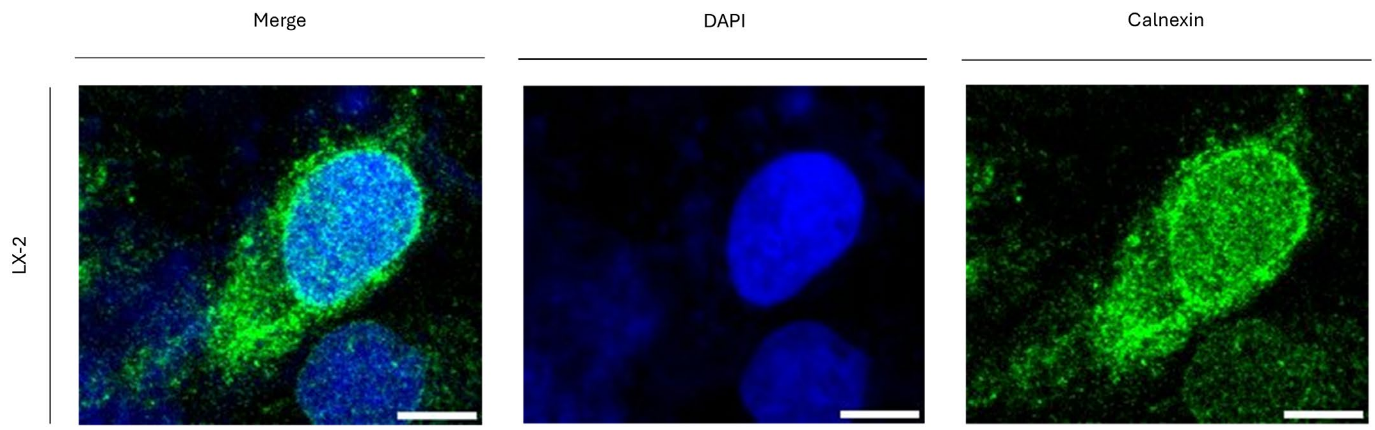

Supplementary Figure S6. Endoplasmic Reticulum visualization in human hepatic/liver, immortalized/"normal", cells. CLSM-mediated immunofluorescence images of LX-2 (human stellate hepatic/liver) cells, presenting the sub-cellular distribution patterning of Calnexin, an Endoplasmic Reticulum (ER) membrane protein and major ER-specific marker. Calnexin is visualized in green colour; and cell nucleus is shown in blue colour (DAPI). Scale bar: 10 μm.

Disclaimer/Publisher's Note: The statements, opinions and data contained in all publications are solely those of the individual author(s) and contributor(s) and not of MDPI and/or the editor(s). MDPI and/or the editor(s) disclaim responsibility for any injury to people or property resulting from any ideas, methods, instructions or products referred to in the content.
